# The Two Main Olfactory Receptor Families in *Drosophila*, ORs and IRs: A Comparative Approach

**DOI:** 10.3389/fncel.2018.00253

**Published:** 2018-08-30

**Authors:** Carolina Gomez-Diaz, Fernando Martin, Jose Manuel Garcia-Fernandez, Esther Alcorta

**Affiliations:** ^1^Department of Functional Biology, Faculty of Medicine, University of Oviedo, Oviedo, Spain; ^2^Department of Cellular Biology, Faculty of Medicine, University of Oviedo, Oviedo, Spain

**Keywords:** chemosensation, receptors, *Drosophila*, ORs, IRs, olfaction, taste

## Abstract

Most insect species rely on the detection of olfactory cues for critical behaviors for the survival of the species, e.g., finding food, suitable mates and appropriate egg-laying sites. Although insects show a diverse array of molecular receptors dedicated to the detection of sensory cues, two main types of molecular receptors have been described as responsible for olfactory reception in *Drosophila*, the odorant receptors (ORs) and the ionotropic receptors (IRs). Although both receptor families share the role of being the first chemosensors in the insect olfactory system, they show distinct evolutionary origins and several distinct structural and functional characteristics. While ORs are seven-transmembrane-domain receptor proteins, IRs are related to the ionotropic glutamate receptor (iGluR) family. Both types of receptors are expressed on the olfactory sensory neurons (OSNs) of the main olfactory organ, the antenna, but they are housed in different types of sensilla, IRs in coeloconic sensilla and ORs in basiconic and trichoid sensilla. More importantly, from the functional point of view, they display different odorant specificity profiles. Research advances in the last decade have improved our understanding of the molecular basis, evolution and functional roles of these two families, but there are still controversies and unsolved key questions that remain to be answered. Here, we present an updated review on the advances of the genetic basis, evolution, structure, functional response and regulation of both types of chemosensory receptors. We use a comparative approach to highlight the similarities and differences among them. Moreover, we will discuss major open questions in the field of olfactory reception in insects. A comprehensive analysis of the structural and functional convergence and divergence of both types of receptors will help in elucidating the molecular basis of the function and regulation of chemoreception in insects.

## Introduction

Detection of chemical cues in the environment is essential for almost all animals to find food, mates, habitats or to avoid predators. Among all invertebrates, insect olfactory systems have extreme sensitivity and discrimination power to detect volatile chemicals related to their food, conspecifics and predators. In this regard, the olfactory system of *Drosophila* resembles the organizational principles of the olfactory system of vertebrates despite being simpler (Stocker, 2001; Vosshall and Stocker, 2007; Touhara and Vosshall, 2009). Thus, in both vertebrates and invertebrates, odorants bind to transmembrane odorant receptors (ORs) expressed in the cilia or dendrites of bipolar olfactory sensory neurons (OSNs). The OSNs send their axons to the brain, where they connect with second-order neurons in the glomeruli of the antennal lobe, in insects, or its homolog in vertebrates, the olfactory bulb. Furthermore, in both types of organisms, each OSN expresses one or very few olfactory molecular receptors, and the axons of OSNs that express the same receptor project to the same glomeruli in both the olfactory bulb and the antennal lobe (Su et al., 2009). These similarities and the fact that *Drosophila* is a widespread model organism in genetics and neurobiological research made its olfactory system an attractive and simple model to study olfaction (see, for example, the recent reviews by Wilson, 2013; Barish and Volkan, 2015; Carraher et al., 2015; Joseph and Carlson, 2015; Fleischer et al., 2018; Grabe and Sachse, 2018; Rimal and Lee, 2018).

In *Drosophila* adults, OSNs are housed inside hair-like structures called sensilla on the surface of the olfactory organs, the third antennal segment and the maxillary palps (Shanbhag et al., 1999). Each sensillum contains 1–4 OSNs (Shanbhag et al., 1999; Joseph and Carlson, 2015). Based on their morphology, sensilla are classified into four types: basiconic, trichoid, intermediate and coeloconic (Shanbhag et al., 1999; Lin and Potter, 2015). Additionally, they differ in regionalization (Shanbhag et al., 1999) and in the substances they detect (de Bruyne et al., 1999, 2001; Yao et al., 2005). These sensilla have pores in the cuticle that allow odorants to diffuse into the sensillum lymph and reach the dendrites of OSNs with the help of odorant binding proteins (OBPs) (Shanbhag et al., 2001; Leal, 2013). Then, odorants are recognized by specific transmembrane protein receptors in the dendrites of the OSNs. In insects, two main families of olfactory receptors have been described, the ORs and the ionotropic receptors (IRs), although a third family, the gustatory receptors (GRs), is involved in carbon dioxide detection (Jones et al., 2007; Kwon et al., 2007). Only one or very few of these olfactory receptors are expressed in each OSN (Vosshall et al., 2000; Benton et al., 2009), similar to what is observed in vertebrates (Buck, 2000). Both receptor families likely form heteromeric complexes between a specific receptor and a co-receptor needed for cellular trafficking and function (Larsson et al., 2004; Neuhaus et al., 2005; Benton et al., 2006, 2009; Silbering et al., 2011). However, although ORs and IRs share characteristics including their role as initial chemosensors for the insect olfactory system, research advances in the last decade have shown that they have distinct evolutionary origins (Robertson et al., 2003; Croset et al., 2010) and have several distinct structural and functional characteristics (Hallem et al., 2004; Benton et al., 2009).

In this review, we will present the research advances of the genetic basis, evolution, structure, functional response and regulation of both OR and IR chemosensory families. For this, we will use a comparative approach emphasizing the similarities and differences among them. Additionally, we will consider key open questions in the field of olfactory reception in insects. Thus, we will help in elucidating the molecular basis of the function and regulation of chemoreception in insects by performing a comprehensive analysis of the structural and functional convergence and divergence of both types of receptors.

## The Molecular Basis of Chemosensation in *Drosophila*: Molecular Structure of ORs and IRs

During the 1990s, the first attempts to discover the chemoreceptors in insects by sequence similarity failed because of their lack of homology with the G protein-coupled (GPCR) ORs of vertebrates (Buck and Axel, 1991) and nematodes (Troemel et al., 1995). Later, using difference cloning and mining of genome databases, a family of proteins with seven transmembrane domains with expression in the OSNs, the OR family, was discovered in *Drosophila* (Clyne et al., 1999b; Gao and Chess, 1999; Vosshall et al., 1999).

These *Drosophila* ORs show no obvious sequence homology with GPCRs and display an inverted topology with an intracellular N-terminus and an extracellular C-terminus (Benton et al., 2006; Lundin et al., 2007; Smart et al., 2008). Structurally, ORs likely form heteromers composed of one odor-specific OR and another member of the OR family, the odorant receptor co-receptor (ORCO; previously known as OR83b) (Larsson et al., 2004; Neuhaus et al., 2005; Benton et al., 2006). ORCO is highly conserved across insect species (Krieger et al., 2003; Pitts et al., 2004; Jones et al., 2005; Smadja et al., 2009), and it is also necessary for the trafficking of ORs to the ciliary membrane *in vivo* (Larsson et al., 2004; Benton et al., 2006).

In contrast to GPCRs, ORs do not have conventional binding sites for G proteins, and several studies have reported that OR-ORCO heteromers expressed in heterologous systems can act as odorant-gated ionotropic channels with ionic permeability to Ca^2+^, Na^+^ and K^+^ (Sato et al., 2008; Smart et al., 2008; Wicher et al., 2008; Nakagawa and Vosshall, 2009). However, one of these studies also reported a metabotropic component that is dependent on G proteins and the cAMP transduction cascade (Wicher et al., 2008). This metabotropic component in the function of the OR-ORCO heteromers has been either supported (Kain et al., 2008; Chatterjee et al., 2009; Deng et al., 2011; Ignatious Raja et al., 2014; Miazzi et al., 2016; Murmu and Martin, 2016) or argued against (Yao and Carlson, 2010) in several studies of G-proteins in genetically modified flies.

Additionally, it has been reported that the activity of ORCO is regulated by phosphorylation via protein kinase C (PKC), which is activated by the inositol 1,4,5-inositol triphosphate/diacyl glycerol (IP3/DAG) signal transduction cascade (Sargsyan et al., 2011). Two hypotheses have been proposed in *Drosophila* to explain these results: (a) it is possible that ORs may be mixed ionotropic-metabotropic receptors (Wicher, 2010); or (b) alternatively, ORs may be metabotropically modulated ionotropic receptors (Nakagawa and Vosshall, 2009). These two hypotheses raise two different putative structures of the odor-gated ionic channel that combine either four or two OR/ORCO heteromers. If, as for the other known superfamilies of ligand-gated ionic channels, the central pore is formed by the combination of four subunits (Carraher et al., 2015), then two putative structures could arise. In the first putative structure, four OR subunits would bind the odorants and produce fast opening of an ionic channel formed by four ORCO subunits. Additionally, the OR subunits interact with G proteins that produce slower metabotropic transduction cascades that regulate the ORCO channel (Wicher, 2010). In the second structure, two OR and two ORCO subunits form the central pore of the ion channel, which would open when a ligand binds the OR subunits and could be regulated by metabotropic transduction cascades (Nakagawa and Vosshall, 2009). In some other insect species, a third hypothesis proposing only metabotropic signal transduction and ORCO functioning as a pacemaker channel controlling membrane potential has been suggested (Stengl, 2010).

While there are still no X-ray crystallography 3D structures for the *Drosophila* ORs, site-directed mutagenesis, resonance energy transfer and structural modeling efforts (Hopf et al., 2015), have started to provide information about the molecular structure of these seven-transmembrane-domain receptors (Carraher et al., 2015). For example, the second extracellular loop has been suggested to form a lid over the binding pocket, which is formed by the extracellular regions of some transmembrane helices, especially the third and to a less extent the sixth and seventh, of the OR subunits (Carraher et al., 2015). In addition, the interaction between the ORCO and OR receptor subunits through the final intracellular loop and the adjacent transmembrane helices might be important for transducing ligand binding into receptor activation (Benton et al., 2006; Kumar et al., 2013). Furthermore, channel gating could be regulated using phosphorylation sites (Sargsyan et al., 2011) and a calmodulin-binding site in the second intracellular loop of the ORCO subunits (Mukunda et al., 2014; Bahk and Jones, 2016).

Ten years after ORs have been identified, in 2009, a new family of olfactory receptors in *Drosophila* was discovered, an ionotropic glutamate receptor (iGluR)-related family of receptors, termed the IRs (Benton et al., 2009). The IRs are formed by an extracellular N-terminus, a highly variable ligand-binding domain with two lobes separated by an ion channel domain, and a short cytoplasmic C-terminus (Benton et al., 2009). Sixteen IRs out of the 66 discovered are expressed in antennal neurons, while the rest, named “divergent IRs”, are expressed in other locations in the body (Benton et al., 2009; Sánchez-Alcañiz et al., 2018).

While most IRs lack glutamate-binding residues, members of the IR family of olfactory receptors show similarities with the iGluRs of vertebrates and are suggested to form ligand-gated ion channels (Benton et al., 2009; Abuin et al., 2011; Rytz et al., 2013). Although X-ray crystallography 3D structures for IRs are not yet available, some protein homology modeling has been performed (Prieto-Godino et al., 2016, 2017). Still, the exact molecular mechanism of IR activation by different specific odorants remains to be shown, as for ORs. Although the molecular structures of ORs and IRs are extremely dissimilar, they show some commonalities. Both ORs and IRs form functional heterodimeric complexes of a receptor and a coreceptor. However, while the OR functional unit consists of the highly conserved co-receptor ORCO and an odorant-specific OR, which provides the complex with its ligand specificity (Benton et al., 2006), IRs show more than one possible co-receptor—IR25, IR8a, IR76b and IR93a—and a specific IR that, as for ORs, gives the complex its odorant specificity (Benton et al., 2009; Silbering et al., 2011; Ai et al., 2013). For both receptor families, the co-receptors are needed both for odor-evoked electrophysiological neuronal responses and receptor trafficking to the ciliary membrane, the sensory compartment where odorant transduction takes place (Benton et al., 2009; Abuin et al., 2011; Silbering et al., 2011; Ai et al., 2013). IRs show reciprocal need for the co-receptor and the ligand-specific IR for dendrite trafficking (Benton et al., 2009; Abuin et al., 2011). Similarly, ORs need ORCO for trafficking to ciliary membranes (Larsson et al., 2004; Bahk and Jones, 2016). The transport of *Drosophila* ORs to and within the dendritic cilia is regulated by the hedgehog (Hh) signal transducer smoothened (Smo) (Sanchez et al., 2016).

Intriguingly, antennal pheromone-sensing neurons in insects show the expression of another membrane-bound element, the sensory neuron membrane protein 1 (SNMP1) (Rogers et al., 1997, 2001; Jiang et al., 2016). SNMP1 is a CD36-related receptor whose involvement in sensing cis-vaccenyl acetate (cVA), a pheromone produced by males in *Drosophila*, has been extensively studied (e.g., Benton et al., 2007; Jin et al., 2008). SNMP1—together with the complex ORCO/OR67d and the odorant/pheromone binding protein, LUSH—is essential for cVA electrophysiological responses but not for trafficking the OR complex to the sensory cilia (Benton et al., 2007). However, SNMP1 might support the functional expression of DmORCO found in mammalian cell culture (Halty-deLeon et al., 2016). Moreover, it has been shown that SNMP1 is important for both rapid activation and termination of the cVA response (Li et al., 2014). Based on homology modeling and structure-function studies, it has been recently proposed that SNMP1 funnels hydrophobic pheromones through a putative ectodomain tunnel from the extracellular fluid to the membrane receptors (Gomez-Diaz et al., 2016). Although some members of the IR family, such as the IR20a clade, have been proposed as pheromone sensors (Koh et al., 2014), no evidence has been found for an equivalent to SNMP1 dedicated to pheromone sensing for the IRs.

Other members of the peripheral sensory system include the OBPs (Vogt and Riddiford, 1981). They are secreted by auxiliary cells in the antenna and show specific sensillar patterns (Shanbhag et al., 2001; Leal, 2013). Although the number of OBP genes in *Drosophila* is similar to the number of ORs and they both show similar patterns of evolution in some species (Kopp et al., 2008), there are also some OBPs found both in taste organs and antennal IR-expressing neurons (Galindo and Smith, 2001; Shanbhag et al., 2001). The specific role and action mechanism of OBPs in olfaction are still under debate; although there are some OBPs, such as LUSH, that have been linked to the detection of odorants by ORs (Xu et al., 2005; Swarup et al., 2011; Gomez-Diaz et al., 2013), no functional evidence has been found for the need of OBPs in IR-expressing neurons.

## The Genetic Basis of OR and IR Protein Families

**Figure 1:**
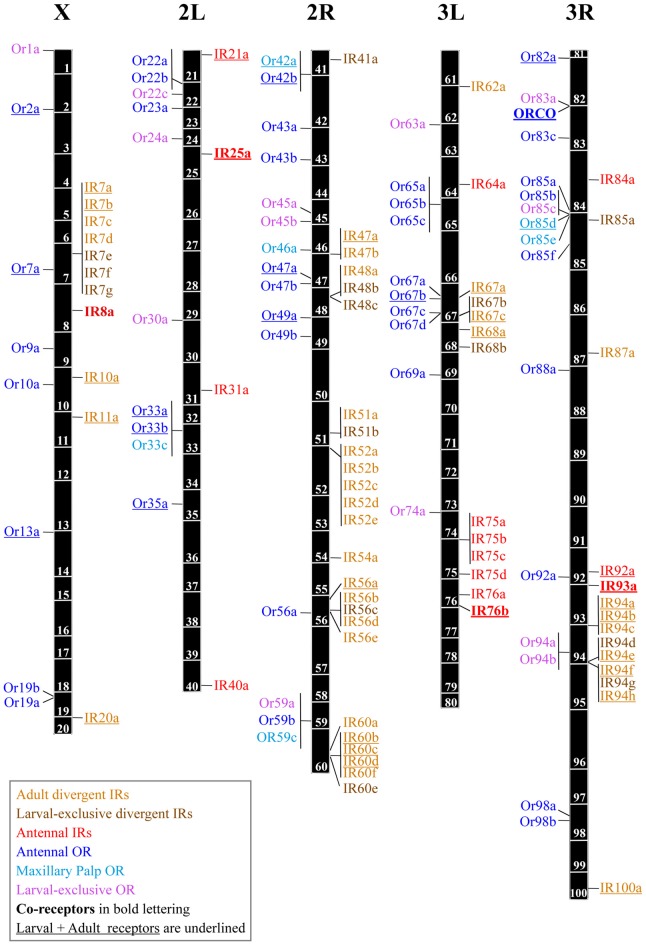
Genomic locations of the odorant receptor (OR) and ionotropic receptor (IR) genes. The five major chromosome arms are drawn to scale, with OR genes shown left and IR genes right of each chromosome arm. Gene locations are based on data from Release 6 of the genome of *D. melanogaster* and the FlyBase database (release FB2018_02; Gramates et al., 2017) The names of the olfactory receptors are color-coded as follows: adult divergent IRs (light brown), larval-exclusive divergent IRs (brown), antennal IRs (red), antennal ORs (blue), maxillary palp ORs (light blue) and larval-exclusive ORs (magenta). Co-receptors are indicated in bold letters. Expression both in adults and larvae is indicated as underlined text (Couto et al., 2005; Fishilevich et al., 2005; Benton et al., 2009; Sánchez-Alcañiz et al., 2018).

The OR protein family is encoded by 60 genes and a few pseudogenes in the *Drosophila* genome. It comprises 62 receptor proteins as Or46a and Or69a each give rise to two proteins by alternative splicing (Robertson et al., 2003). Some of the OR genes are clustered together in groups of two or three, probably because they are recent duplications, but most of them are widely dispersed in the genome (Robertson et al., 2003) (Figure 1). The IR receptor family is extremely divergent, showing an overall amino acid sequence identity of 10%–70%. Similar to the ORs, these genes are distributed throughout the *Drosophila* genome, many as individual genes, although some form cluster arrays of few genes as e.g., in cytological regions 7 and 52 (Benton et al., 2009) (Figure 1).

Expression studies of OR genes have shown that 45 members of this family are present in the adult antenna and maxillary palp, while 25 are expressed in the larval olfactory system. Some ORs are expressed in both developmental stages, and some are exclusive to one, either larvae or adults (Couto et al., 2005; Fishilevich et al., 2005). In the antenna, each OR is expressed in 2–50 OSNs (Clyne et al., 1999b; Vosshall et al., 1999, 2000) in a stereotyped sensillar map in which each sensilla subtype is characterized by the expression of one or few ORs in each of the OSNs that innervate it. ORs are almost uniquely expressed in neurons housed in single-walled sensilla, such as basiconic, trichoid and intermediate sensilla (Shanbhag et al., 1999; Lin and Potter, 2015), while IRs are expressed in double-walled coeloconic sensilla (Benton et al., 2009; Silbering et al., 2011, 2016). Thus, 18 sensilla types house 39 OSN classes that express OR members with ten basiconics, two trichoids, three intermediates in the antennae as well as three basiconic sensilla types on the palps (Couto et al., 2005; Lin and Potter, 2015) Although ORs and IRs are expressed on different sensory lineages in the antenna, there is one exception: in ac3 sensilla, IR76b is coexpressed with OR35a/ORCO (Benton et al., 2009). Additionally, one of the four OSNs of the ab1 basiconic sensilla expresses two GRs, Gr21a and Gr63a, both dedicated to CO2 sensing (Jones et al., 2007; Kwon et al., 2007). Similar to vertebrates, each OSN usually expresses only one type of specific OR (and ORCO), although there are some exceptions in which two or three ORs are expressed in the same OSNs, such as ab5 sensilla, where OR33b and OR47a, both present in larvae and adult, are expressed together (Fishilevich and Vosshall, 2005; Goldman et al., 2005).

Genomic analysis in *Drosophila* has revealed 66 IR genes, including nine putative pseudogenes (Benton et al., 2009; Croset et al., 2010). Extensive effort in expression analysis has shown that antennal neurons express 16 IRs, most of which are in neurons housed in coeloconic sensilla. Four IRs, IR20a, IR40a, IR64a and IR93a, are expressed not in the coeloconic neurons but in the arista and sacculus neurons (Benton et al., 2009; Silbering et al., 2016). By using transgenic reporters, it was revealed that out of the 44 non-antennal IRs, 32 were expressed in larvae and 27 in adults, where they were found in various organs, such as the antennae, labella, pharynx, legs and wings (Joseph and Carlson, 2015; Sánchez-Alcañiz et al., 2018). Similar to ORs, some of them are specific to either larvae or adults, such as the IR52 clade, expressed in foreleg taste neurons, which has been related to mating behavior (Koh et al., 2014). Neurons housed at coeloconic sensilla express from two to four IRs (Benton et al., 2009). Additionally, IRs do not seem to be expressed in the secondary olfactory organs, the maxillary palps, which only contain OR-expressing neurons.

The expression studies carried out on both types of receptors have also permitted the generation of a complete projection map of the axons of the OSNs to the 52 glomeruli of the antennal lobe in the brain, showing that every OSN that expresses a particular olfactory receptor sends axonal projections to the same glomerulus (Couto et al., 2005; Silbering et al., 2011; Grabe and Sachse, 2018). There is a spatial organization in which the afferents from the OSNs innervating each type of sensilla project to glomeruli in the same location in the antennal lobe. Thus, OSNs in the antennal basiconic sensilla project to the medial region of the antennal lobe, palp basiconic sensilla to the central-medial region, antennal trichoid sensilla to the lateral anterior region, and antennal coeloconic sensilla to the posterior region (Couto et al., 2005; Silbering et al., 2011). Therefore, projections of OR- and IR-expressing neurons are segregated although interconnected in the antennal lobe (Silbering et al., 2011).

The genetic control of the stereotyped expression of the ORs of both families in the OSNs is still under study. There is a relationship between the expression of olfactory receptors and the zonal localization of sensillum types/subtypes. Expression of a given receptor is restricted to an OSN class that is located in a particular sensillum subtype, and thus, all OSNs form a sensory map on the antenna (Vosshall et al., 1999, 2000; Couto et al., 2005; Benton et al., 2009; Silbering et al., 2011). Each sensillum subtype houses stereotypical clusters of 1–4 OSN identities that arise through asymmetric divisions from a single multipotent sensory organ precursor (SOP; Rodrigues and Hummel, 2008).

The adult olfactory organs develop from the larval antennal imaginal disc, where the various morphological types of sensilla arise in the pupae due to the action of a combination of proneural and helix–loop–helix transcription factors (TFs) (Fuss and Ray, 2009). Thus, the TF *atonal* is necessary for the development of the antennal coeloconic and palp basiconic sensilla (Gupta and Rodrigues, 1997), while *amos* and *lozenge* are required for the antennal basiconic and trichoid sensilla (and the few intermediate sensilla; Gupta et al., 1998; Goulding et al., 2000; zur Lage et al., 2003). In the antennal disc, the differential expression of *Dachshund*, *Rotund*, *BarH1/H2*, *Bric-à-brac* and *Apterous* patterns the antennal disc into seven concentric rings (Li et al., 2016; Hsieh et al., 2017). Each concentric ring will determine a subset of the subtypes of sensilla in the antenna; for example, the innermost ring determines the SOPs for the at2, ac3, ab2, ab3, ab4, ab6 and ab8 sensilla (Li et al., 2016; Hsieh et al., 2017). Finally, in each of these rings, the determined SOPs will develop into a particular sensilla subtype, and each OSN they house will specifically express one or few ORs (Barish and Volkan, 2015). In contrast to insect OR-expressing neurons, where most of them expresses a unique odorant-specific receptor type, and the co-receptor ORCO, IR-expressing neurons do show a more complicated receptor choice specification with some neurons expressing more than two IRs, needed for its functional response (Benton et al., 2009), but any information on the IR specification remains elusive. Conversely, in OR-expressing neurons, various studies have implicated several TFs in the OR choice specification of each OSN (Martin et al., 2013). Thus far, five TFs have been implicated in the regulation of the ORs expressed in the palps (Clyne et al., 1999a; Ray et al., 2007; Tichy et al., 2008; Bai and Carlson, 2010; Song et al., 2012), while in the antennae, at least nine TFs are involved in the control of OR expression (Jafari et al., 2012; Song et al., 2012). Recently, a genetic immortalization method has been used to elaborate a fate map of all olfactory lineages and to identify *Pointed*, a E26 transformation-specific transcription factor (ETS) family member, as a determinant of the Or67d pheromone-sensing neuron development (Chai et al., 2018). Additionally, in a sensillum, a cluster of OSNs is asymmetrically differentiated from a single SOP into two classes in a manner dependent on differential Notch activity in their sibling precursors. In this way, Notch-ON and Notch-OFF specify olfactory receptor expression and axonal targeting of the different OSNs housed in a single sensillum (Endo et al., 2007). This pathway is dependent on the co-repressor Atrophin, which regulates Histone 3 acetylation to determine the OR expressed in any OSN (Alkhori et al., 2014). Apart from the TFs that regulate the expression of the ORs, mutagenesis of the upstream regulatory sequences of four OR genes has identified particular sequence motifs that act positively or negatively to dictate expression in the proper subset of OSNs (Miller and Carlson, 2010).

## Evolution of Both Families of Chemoreceptors

Although the IR family is related to the iGluRs that mediate synaptic communication in vertebrate and invertebrate nervous systems (Benton et al., 2009), the insect OR receptors are not related to the ORs found in vertebrates and nematodes and have evolved independently (Robertson et al., 2003; Benton et al., 2006).

In the case of the OR family, via comparative genomic and transcriptomic analyses, several related but highly divergent genes have been found in many insect genomes (Grosse-Wilde et al., 2011; Kanost et al., 2016; de Fouchier et al., 2017), ranging from the seven ORs found in the human body louse (Pelletier et al., 2015), 79 ORs in the malaria mosquito (Fox et al., 2001; Hill et al., 2002), 163 in the honey bee (Robertson and Wanner, 2006), 256 in the red flour beetle (Engsontia et al., 2008; Dippel et al., 2016), to the more than 350 ORs found in some ant species (Zhou et al., 2012). In social insects, the chemosensory protein repertoire shows an OR-specific expansion (up to 450 OR candidates found in antennal transcriptomes and genome-wide analysis) that does not seem to affect the IR or the GR families (Robertson and Wanner, 2006; Zhou et al., 2012; McKenzie et al., 2014; Oxley et al., 2014; Pitts et al., 2017). This expansion has been hypothesized to be linked to the strong diversification of flowering plants as food sources and to the enhanced needs for discrimination between nestmates and non-nestmates and for reproductive division of labor in social insects, although direct evidence for this in different species is still scarce (e.g., Sharma et al., 2015; Pask et al., 2017). Comparative phylogenetic analyses of these expanded ORs have allowed the identification of some OR subfamilies, as e.g., pheromone receptors, and to study their evolutionary origin and expansion in insect lineages (Missbach et al., 2014; Koenig et al., 2015; de Fouchier et al., 2017). In the case of ORCO, highly conserved homologs have been found in several insect orders, such as Lepidoptera, Diptera, Coleoptera, Hymenoptera, Hemiptera and Orthoptera (Krieger et al., 2003; Pitts et al., 2004; Smadja et al., 2009; Yang et al., 2012). However, in some cases, when the genomes of primitive not winged insects have been investigated, either no OR or ORCO was found (as in one member of the order Archaeognatha), or only a few gene homologs of ORCO were identified (as a species of the order Zygentoma; Missbach et al., 2014). Additionally, in the crustacean *Daphnia pulex*, which shares a common ancestor with insects, despite the fact that members of the GR family were identified, no ORs were found (Peñalva-Arana et al., 2009). Likewise, ORs are absent in the genomes of other arthropods, such as spiders (Vizueta et al., 2017). These data suggest that the OR family is exclusive to insects and probably evolved when insects developed flight, with the evolution of ORCO first and the other OR subfamilies later (Missbach et al., 2014). It is thought that both the ORs and GRs are part of a superfamily of chemosensory receptors (Robertson et al., 2003) and that the OR family evolved from the GR family, which can be found in all arthropods, when the insects became terrestrial organisms and started to fly.

In contrast to the insect-specific origin of the OR family, comparative genomic analysis across many animal groups has revealed an ancient Protostome origin for the IR family (Croset et al., 2010; Rytz et al., 2013). While antennal IRs are conserved and show orthologs in many different insect species, the genomic analysis of non-antennal IRs, originally named “divergent IRs”, indicates a great expansion in Diptera and shows unclear orthologous relationships in other insects, forming phylogenetic species-specific clades across insects. It has been suggested that the enormous expansion of this chemosensory family arose from non-allelic homologous recombination and retroposition (Croset et al., 2010). Most IRs exist as single-copy highly conserved orthologs, but there are some cases where non-allelic homologous recombination and ancient duplication events played a large role in IR evolution, as is the case for the IR75 cluster (Croset et al., 2010).

Olfactory receptor families contain various pseudogenes. In *Drosophila* *sechellia*, IR75a encodes an expected pseudogene, with a premature stop codon, but it has been shown that it is a “pseudo-pseudogene”, meaning that the receptor remains functional due to translational read-through of the premature termination codon (Prieto-Godino et al., 2016), although the exact mechanism of the read-through remains unknown. Another IR receptor, IR31a, showed characteristics of this pseudo-pseudogenization along with OR35a (Prieto-Godino et al., 2016), but whether this is a common feature also in the OR family remains to be shown.

Chemoreceptor families in *Drosophila* are extremely useful models for studying how selection acts over organisms in a changing environment because they show rapid adaptation over short timescales, which seems to be a function of relaxed constraints (Arguello et al., 2016). For example, *Drosophila sechellia* is attracted to hexanoic acid, present in the noni fruit, while *D. melanogaster* is not. This shift in preference is mainly driven by a single amino acid change in the IR75b protein, which together with some changes in the promoter and trans-acting loci, tunes this receptor in *D. sechellia* to hexanoic acid (Prieto-Godino et al., 2017), allowing this species to adapt to its specific ecological niche.

By analysis of genome-wide data, including single-nucleotide polymorphisms (SNPs), copy number variants (CNVs) and small insertions and deletions (indels), of chemosensory families from various *Drosophila melanogaster* populations (from ancestral-like African populations to subsequent populations that inhabit different niches) and comparison with other large families, it has been demonstrated that chemosensory receptors do not show high rates of adaptive divergence between species but show genome-wide signals of recent selection within *D. melanogaster* (Arguello et al., 2016). Additionally, they display patterns of adaptive mutations that could predict diverse effects on protein function (Arguello et al., 2016).

## Functional Regulation of Neuronal Response

**Figure 2:**
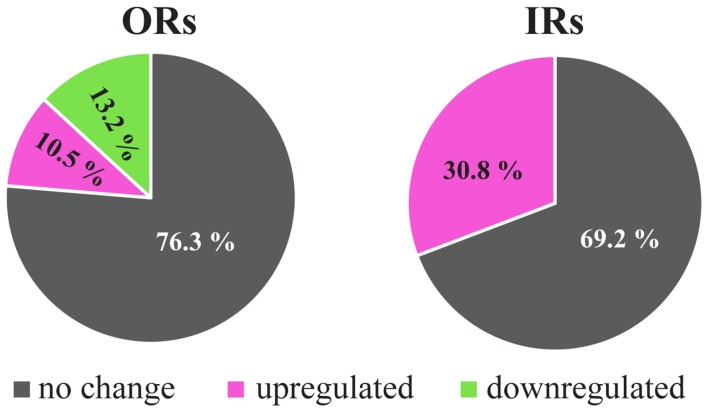
Changes in the antennal expression levels of OR and IR genes in response to high temperature. Percentage of detected genes that show up- and down-regulated gene expression under heat treatment condition (shifting from 21°C to 30°C) using Affymetrix microarrays. Only significant changes with a false discovery rate (FDR) <0.1 were considered. Data adapted from Riveron et al. (2013).

The responses of the OSNs could be modified by several external and internal factors that affect the olfactory receptor function at various levels, from genetic expression to functional interaction. For example, the environmental temperature affects olfactory behavior in *Drosophila* (Riveron et al., 2009) and also modifies antennal electrical responses of OR-expressing OSNs, as shown in electroantennograms (EAGs) and single-sensillum recordings (SSRs) (Martin et al., 2011). Correspondingly, in microarray transcriptomic studies using third antennal segments of high-temperature-acclimated flies, there were changes in the expression levels of several ORs and IRs (Riveron et al., 2013). Although high temperature produced significant overexpression only in four out of the 16 antennal IRs, the same tendency was shown in the rest of the IRs analyzed (Figure 2) (Riveron et al., 2013). However, for the ORs, significant changes for nine members of the family were found, four of them displaying overexpression and the other four and ORCO showing downregulation (Figure 2).

Additionally, internal signals can regulate the responses of OSNs expressing ORs. For example, in a lepidopteran, the crepuscular hawk moth, *Manduca sexta*, there is circadian control through octopamine (OA) over the olfactory metabotropic transduction of pheromones (Schendzielorz et al., 2015). OA could act on ORCO, which has been suggested to be a hormone-controlled pacemaker channel controlling spontaneous activity, threshold and temporal resolution of pheromone detection (Stengl, 2010; Stengl and Funk, 2013). In this species, no evidence of ORCO-based ionotropic signal transduction cascade has been found (Nolte et al., 2013, 2016).

Furthermore, the internal amino acid state can modulate yeast taste neurons. A common subset of the population of IR76b- and IR25-expressing neurons in the proboscis is required for yeast sensing (Steck et al., 2018). The response of these gustatory receptor neurons (GRNs) is directly modulated by the internal amino acid state, while the reproductive state modulates yeast feeding downstream of the receptor neurons (Steck et al., 2018).

Olfactory neuronal responses driven by both types of chemoreceptors can be differentially regulated. For example, the OR-expressing OSNs strongly adapt to odors in electrophysiological recordings of the whole antenna (electroantennograms, EAGs) (Störtkuhl et al., 1999), individual sensilla, (single-sensillum recordings, SSRs) (Nagel and Wilson, 2011; Martelli et al., 2013) or single OSNs (whole-cell patch clamp) (Cao et al., 2016). In contrast, IR-expressing OSNs showed no adaptation both in sensillum recordings (Abuin et al., 2011) and in whole cell patch clamp (Cao et al., 2016), indicating that the two types of neurons might use distinct odor transduction mechanisms. The adaptation in the OR/ORCO receptors seems to be mediated by odorant-induced phosphorylation changes of the serine 289 of ORCO (Guo and Smith, 2017). Additionally, there is a decrease in the spike amplitude in the SSR of OSNs expressing ORs during odor stimulation that has been related to its concentration (Martin and Alcorta, 2016).

Moreover, some effort has been made in describing the functional dynamics and latencies of the responses to odors in OR-expressing OSNs in *Drosophila* (Martelli et al., 2013) because a pure ionotropic response does not involve amplification and is believed to be faster than a metabotropic response. Although direct evidence for response latencies in ORs and IRs in *Drosophila* is still missing, these latencies have been investigated in EAGs of other insect species, such as the orange spotted cockroach (*Blaptica dubia*), hissing cockroaches (*Gromphadorhina portentosa*), locusts (*Schistocerca americana*), honey bees (*Apis mellifera*) and moths (*Manduca sexta*), showing latencies as short as 2 ms (Szyszka et al., 2014).

## Functional Profiles of ORs and IRs

From the perspective of sensory modalities that involve these two peripheral sensory systems, it seems that the OR receptor family is exclusively used in olfaction, while the IR family covers both chemosensory modalities, olfaction and taste, and even non-chemosensory ones (Table 1).

**Table 1:** Comparison of the main characteristics and functional properties of OR and IR-expressing neurons.

OSN type	Co-receptor	Latency of response	Adaptation	Adult sensory organ	Sensory modality
**OR-expressing neuron**	Yes (ORCO) (Larsson et al., 2004; Benton et al., 2006)	2 ms (Szyszka et al., 2014)	Yes (Störtkuhl et al., 1999; Nagel and Wilson, 2011; Martelli et al., 2013; Cao et al., 2016)	**Antenna** Basiconic, Trichoid and Coeoloconic sensilla* **Palps** Basiconic sensilla (Couto et al., 2005)	**Olfaction** Food odors (Hallem et al., 2004; Hallem and Carlson, 2006) Pheromones (Benton et al., 2007; Kurtovic et al., 2007; van der Goes van Naters and Carlson, 2007)
**IR-expressing neuron**	Yes (IR25a, IR8a, IR76b, IR93a) (Benton et al., 2009; Abuin et al., 2011; Sánchez-Alcañiz et al., 2018)	-	No (Benton et al., 2009; Cao et al., 2016)	**Antenna**, Coecolonic sensilla, sacculus (Benton et al., 2009)	**Olfaction** Food odors (Benton et al., 2009; Abuin et al., 2011; Silbering et al., 2011) Pheromones (Koh et al., 2014)
				**Labella Pharynx** Labral Sense Organ Dorsal Cibarial Sense Organ Ventral Cibarial Sense Organ **Leg Wing margin** (Koh et al., 2014; Sánchez-Alcañiz et al., 2018)	**Taste** Polyamines (Hussain et al., 2016) Salt (Zhang et al., 2013; Lee et al., 2017, 2018) Aminoacids (Croset et al., 2016; Ganguly et al., 2017) Fatty acids (Ahn et al., 2017; Tauber et al., 2017) Sugar (Joseph et al., 2017) Carbonation (Sánchez-Alcañiz et al., 2018)
				**Antenna**, sacculus and arista (Benton et al., 2009)	**Hygrosensation** (Enjin et al., 2016; Knecht et al., 2016, 2017) **Temperature sensing** (Knecht et al., 2016; Ni et al., 2016)

*Only one OR has been ascribed to coeloconic sensilla.

Several studies have been carried out to establish the odorant response profiles of OR receptors using electrophysiological measurements, such as SSRs, obtained either by their ectopic expression in an empty neuron (Hallem et al., 2004; Hallem and Carlson, 2006) or directly in native OSNs (Clyne et al., 1997; de Bruyne et al., 1999, 2001). These studies involved panels of 100 odorants at most, although a computer simulation study with 240,000 odorants was partially validated in functional assays (Boyle et al., 2013). Likewise, similar studies have been performed to determine the odorant response profiles of the IR receptor family (Benton et al., 2009; Abuin et al., 2011; Silbering et al., 2011). All available *Drosophila* odorant response data have been combined to a single consensus response matrix linking odorants to olfactory receptors in the DoOR database (Galizia et al., 2010; Münch and Galizia, 2016).

Both chemosensory families are involved in food odor sensing, detecting a vast array of chemicals. While ORs are highly tuned to esters and alcohols (Hallem et al., 2004; Hallem and Carlson, 2006), IRs are highly tuned to amines and acids (Benton et al., 2009; Abuin et al., 2011; Silbering et al., 2011; Min et al., 2013). Usually, the ORs are broadly tuned to several compounds, while the IRs are more narrowly tuned to a few compounds (Silbering et al., 2011).

While some ORs, especially those expressed in trichoid sensilla, have been shown to be responsive to pheromones (Benton et al., 2007; Kurtovic et al., 2007; van der Goes van Naters and Carlson, 2007; Stengl, 2010), only a few IRs have been linked to pheromone sensing either indirectly (Grosjean et al., 2011) or through the IR20a clade (Koh et al., 2014). Neurons that express members of the IR20a clade are mostly located in the proboscis, pharynx, legs and wing margin of *Drosophila*. They send their axonal projections to taste centers in the brain that do not overlap with bitter-sensing neurons. Some of these members are activated by odors from conspecific females and are adjacent to a neural circuit for sexual behavior, the fru+ neurons (Koh et al., 2014). Additionally, IR52c and IR52d show sexually dimorphic expression in leg taste neurons (Koh et al., 2014), but their specific ligands are still unknown.

Some other sensory modalities, such as taste, while multimodal in most cases, seem to be exclusively mediated by GRs, IRs or a combination of both (Vosshall and Stocker, 2007; Liman et al., 2014; Sánchez-Alcañiz et al., 2018). To date, there is no evidence involving ORs in taste sensation. Using transgenic reporters, it has recently been shown that most IRs are expressed in diverse populations of peripheral sensory neurons of gustatory organs in both larvae and adults (Sánchez-Alcañiz et al., 2018). In general, taste seems to require the co-receptors IR25a and IR76b but not IR8a (Sánchez-Alcañiz et al., 2018). In fact, it has been shown that IR25a and IR7b are necessary in female sour-detecting GRNs for oviposition preference in acid-containing food (Chen and Amrein, 2017).

In *Drosophila*, long-range attraction to polyamines, pungent-smelling compounds required in numerous cellular and organismal processes, is mediated by IR76b and IR41a, while short-range attraction, which stimulates egg-laying behavior in polyamine rich-medium, seems to be a multimodal stimulus sensation mediated by IR76b and GR66a bitter-receptor neurons (Hussain et al., 2016). This mechanism seems highly conserved, as it is also found in mosquitoes (Hussain et al., 2016).

Flies use GRNs to respond to different concentrations of salt (Vosshall and Stocker, 2007; Liman et al., 2014). While attractive at low concentrations, salt can be harmful at higher concentrations. Strikingly, a highly conserved IR in insect genomes, IR76b, was shown to be a leak Na^+^ channel that detects low salt and drives the salt-induced attractive pathway, while other GRNs would drive salt-aversive behavior (Zhang et al., 2013). This system could act as a bimodal switch for behavioral salt attraction and aversion (Zhang et al., 2013). Recent reports on Na^+^ sensing in *Drosophila* showed that IR76b-sensing GRNs in both L- and S-bristles are required for repulsion (Lee et al., 2017), contrary to the previous idea of IR76b directing only attraction to low Na^+^ (Zhang et al., 2013). More research will be needed to elucidate this open question. In addition to Na^+^ sensing, excessive Ca^2+^ taste avoidance is also important for avoiding toxic levels of this mineral in the food. This avoidance requires three members of the IR family—IR25a, IR62a and IR76b—expressed in GRNs in the labella, although the ectopic expression of these three elements is not sufficient to confer Ca^2+^ sensitivity, indicating that some other elements are needed (Lee et al., 2018).

Little evidence of a functional role for IRs has been gathered in larvae of *Drosophila*, but recently, it has been shown that larvae lacking IR76b displayed highly reduced behavioral attraction to some amino acids, while those lacking IR25a show no effect in attraction to them (Croset et al., 2016). Using functional imaging, it was shown that only a subset of IR76b-expressing gustatory neurons respond to some amino acids. In these IR76b-expressing cells, increases in calcium levels were observed upon presentation of nine amino acids; however, this subset did not correspond precisely with the stimuli that trigger preference behavior (Croset et al., 2016). These nine amino acids also elicited responses in IR60c-expressing neurons, although these neurons are not required for amino acid preference but rather mediate, together with IR76b, feeding suppression by high concentrations of amino acids (Croset et al., 2016). In adults, IR76b has been postulated as necessary for the post-mating female preference for amino acids by tarsal taste neurons (Ganguly et al., 2017). Additionally, it was suggested that IR20a blocks the IR76b salt-sensing activity and facilitates a mutually exclusive role of IR76b in both salt and amino acid sensing. Co-expression of IR20a confers amino acid sensitivity to sweet-sensing neurons but not to L-type sensilla (Ganguly et al., 2017). Thus, indicating either that some other factor is needed or that the switching mechanism mediated by IR20a could be replaced.

Although little is known about fatty acid detection in insects, it has recently been linked to the IR family of chemoreceptors in *Drosophila* (Ahn et al., 2017; Tauber et al., 2017). By using either Ca^2+^ imaging in sweet-sensing GRNs on tarsal sensilla preparations or behavioral assays (Proboscis extension response, PER) using IR25a and IR76b mutants, the requirement of IR25a and IR76b in fatty acid detection was shown (Ahn et al., 2017). Moreover, both RNAi knockdown of IR56d in sweet-sensing neurons (Ahn et al., 2017) and functional imaging (Tauber et al., 2017) linked this receptor to their detection. Neurons that co-express Gr64f and IR56d are activated by medium-chain fatty acids being sufficient for reflexive feeding responses (Tauber et al., 2017). Fatty acids also elicit responses in bitter-sensing GRNs, but their molecular basis remains unknown (Ahn et al., 2017).

A healthy metabolism requires the control of sugar consumption. In *Drosophila*, it has been shown, by an optogenetic approach, that overconsumption of sugar could be avoided by activation of a circuit that inhibits sucrose feeding depending on IR60b (Joseph et al., 2017). IR60b is co-expressed in a neuron in the pharynx together with IR94f and IR94h but not with any sweet-sensing GRs (Joseph et al., 2017), although the roles of IR94h and IR94f remain elusive. While alternative explanations for the role of IR60b have been hypothesized (Szyszka and Galizia, 2018), they need further experimental analysis.

Carbonation, a non-nutritious product of microbial fermentation, has been shown to be detected in *Drosophila* by IR56d-expressing taste neurons together with IR25a and IR76b co-receptors (Sánchez-Alcañiz et al., 2018). Using transgenic reporters, it has been shown that IR56d is expressed in two different neuronal populations: the one in the taste pegs is dedicated to carbonation and fatty acid detection (but not activated by sucrose), while the other one, in taste bristles, is dedicated to sugar and fatty acid sensing (Tauber et al., 2017; Sánchez-Alcañiz et al., 2018). Although carbonation is modestly behaviorally attractive in an IR56d-dependent manner, IR56d seems to be necessary but not sufficient for this attraction (Sánchez-Alcañiz et al., 2018).

In conclusion, different cell-specific IR subunit combinations seem to be the basis for different taste qualities.

Although not the topic of this review, it should be mentioned that very recently, it was discovered that the IR family of receptors, unlike the OR family, covers other sensory modalities beyond chemosensation, such as hygrosensation (Enjin et al., 2016; Knecht et al., 2016, 2017) and temperature sensing (Knecht et al., 2016; Ni et al., 2016) in both adults and larvae (Sánchez-Alcañiz et al., 2018). Moreover, IR25 has been proposed as a temperature sensor that impacts the temperature-dependent resetting of the circadian clock (Chen et al., 2015).

## Discussion and Closing Remarks

Despite the recent increase in knowledge about the main receptor families in olfaction in *Drosophila* (see reviews by Wilson, 2013; Carraher et al., 2015; Joseph and Carlson, 2015; Fleischer et al., 2018; Rimal and Lee, 2018), there are still many open questions that remain to be answered.

For example, finding the ligands for orphan receptors in both chemosensory families will shed light on the different modalities that they subserve. Additionally, experiments identifying the transduction mechanisms used by the two types of chemoreceptors will also help understanding the exquisite sensitivity and specificity of these receptors. Also, accurate X-ray crystallographic structures will help in solving some of these standing issues such as the exact composition of olfactory receptor heteromers or their ligand binding sites.

Although this review has been focused mainly on adult *Drosophila*, these two families of receptors are also present in larvae. However, few studies focus on larval chemosensory modalities. Twenty-five members of the OR family are expressed in the dorsal organ, the olfactory receptor organ in larvae (Fishilevich et al., 2005) and several studies have characterized their olfactory responses via behavioral tests (Fishilevich et al., 2005; Gomez-Marin et al., 2011) and electrophysiology measures (Hoare et al., 2008; Mathew et al., 2013). As we already mentioned, recent studies on larval IRs (Croset et al., 2016; Sánchez-Alcañiz et al., 2018) have shown their involvement in different taste modalities. Because there are both larval-specific ORs and larval-specific IRs, it could be hypothesized that there might be some larval-exclusive sensory modalities adaptations that have not been investigated yet. Further research on both families of larval chemosensory receptors will be needed to answer this question.

Importantly, much evidence of both expression and functional roles is coming from the use of Gal-4 lines, which are a extremely useful tool in the field but also show some caveats. Surely, the generation of more knockout mutants for the different receptors will answer some of the controversies caused by the caveats of using RNAi knockdown strategies.

Another question that will be addressed in the future is that the most ecologically relevant ligands may not have been found yet. Olfactory receptors are considered narrowly or broadly tuned based on analysis of ligands that may not be relevant at all for the fly (Bohbot and Pitts, 2015). Few works have linked olfactory ecology to structural and regulatory genetic changes in the chemoreceptor families (Prieto-Godino et al., 2017), but in upcoming years, new genome-editing technologies and the advancement of next-generation sequencing in insect species other than *Drosophila* will shed light on the function and evolution of both the OR and IR families (Arguello and Benton, 2017), and such work will have repercussions for controlling pests and diseases transmitted by insect vectors (van der Goes van Naters and Carlson, 2006; Crava et al., 2016; Benton, 2017).

In this review, we have focused on the main peripheral chemosensory systems at the receptor level (Table 1), but the interaction between OR- and IR-related circuits in both first relay and higher processing brain centers (Grabe and Sachse, 2018) is mainly unexplored and of outstanding interest for elucidating the behavioral output of the individual to chemical cues.

## Author Contributions

All the authors listed made substantial contributions to this review. CG-D, FM and EA drafted the initial manuscript. All authors critically read, corrected and approved the final version of the manuscript.

## Conflict of Interest Statement

The authors declare that the research was conducted in the absence of any commercial or financial relationships that could be construed as a potential conflict of interest.

## Abbreviations

CNVs: copy number variants
cVA: cis-vaccenyl acetate
ETS: E26 transformation-specific transcription factor
GR: gustatory receptor
GRN: gustatory receptor neuron
Indels: small insertions and deletions
IR: ionotropic receptor
OBP: odorant binding protein
OR: odorant receptor
ORCO: odorant receptor co-receptor
OSN: olfactory sensory neuron
SNMP1: sensory neuron membrane protein 1
SNPs: single-nucleotide polymorphisms
SSR: single-sensillum recording
TF: transcription factor.
